# Impact of Corticosteroid-Free Regimen on Interstitial Fibrosis Following Kidney Transplantation

**DOI:** 10.1016/j.ekir.2025.04.004

**Published:** 2025-04-06

**Authors:** Simon Ville, Karine Renaudin, Lionel Rostaing, Morgane Pere, Nassim Kamar, Christophe Legendre, Emmanuel Morelon, Elisabeth Cassuto-Viguier, Christophe Mariat, Antoine Durrbach, Matthias Buchler, Laure-Hélène Noel, Eric Thervet, Vannary Meas-Yedid, Diego Cantarovich

**Affiliations:** 1 Institut de Transplantation Urologie Néphrologie (ITUN), CHU Nantes, Nantes, France; 2 Centre de Recherche en Transplantation et Immunologie UMR1064, INSERM, Université de Nantes, Nantes, France; 3 Department of Pathology, CHU de Nantes, Nantes, France; 4 Service de Néphrologie, Hémodialyse, Aphérèses et Transplantation Rénale, CHU Grenoble-Alpes, Grenoble, France; 5 CHU de Nantes, Direction de la recherche, Plateforme de Méthodologie et Biostatistique, Nantes, France; 6 Néphrologie et transplantation d'organes, CHU Rangueil, Toulouse, France; 7 Université de Paris, Service de Néphrologie-Transplantation, Paris, France; 8 Service de transplantation, hôpital Édouard-Herriot, Lyon, France. Université de Lyon-1, France. Unité Inserm U1111, France; 9 Service de néphrologie, dialyse, transplantation rénale, hôpital Pasteur 2, Nice, France; 10 Department of Nephrology, Dialysis and Renal Transplantation, Centre Hospitalier Universitaire de Saint-Étienne, Saint-Etienne, France; 11 Hôpital Henri Mondor, Service de Néphrologie, Assistance Publique-Hôpitaux de Paris, Creteil, France; 12 Service de Néphrologie-HTA, Dialyses, Transplantation Rénale, Hôpital Bretonneau Et Hôpital Clôcheville, CHU Tours, Tours, France; 13 Department of Pathology, Necker Hospital, Paris France; 14 Nephrology Department, European Georges Pompidou Hospital, Assistance Publique-Hôpitaux de Paris, Paris, France; 15 BioImage Analysis Unit, Pasteur Institute, Paris, France

**Keywords:** acute rejection, fibrosis, kidney transplantation, steroid avoidance

## Abstract

**Introduction:**

In kidney transplantation, concerns remain about whether corticosteroids (CS) avoidance could favor interstitial fibrosis (IF) and its progression. We conducted a multicenter randomized noninferiority clinical trial to evaluate the histopathological progression of IF using an innovative automated method in patients receiving CS or not.

**Methods:**

Low immunological risk recipients of a kidney allograft for whom an analyzable biopsy was available at implantation were randomly assigned to receive a CS-free regimen (CS−) or a standard CS tapering regimen (CS+). All patients received induction therapy with basiliximab, and conventional maintenance therapy. The primary outcome was the difference in the percentage change of IF between the baseline and the 1-year protocol biopsy with a prespecified 10% noninferiority margin.

**Results:**

A total of 108 patients were analyzed in the full analysis set (FAS) population as follows: 52 patients in the CS+ group and 56 patients in the CS− group. Complete avoidance of CS was reached in 36 (64%) CS− patients (per-protocol [PP] population). In the FAS population, the mean percentage of IF at implantation was 19.5% ± 7.9% in the CS− group (*n* = 51) and 17.9% ± 7.8% in the CS+ group (*n* = 49; *P* = 0.3), and 25.9% ± 11% (*n* = 43) and 21.5% ± 11.2% (*n* = 39; *P* = 0.03) at 1 year. Considering the difference in IF change, the CS− group was noninferior to the CS+ group neither in the FAS and PP population: 4.45% 95% confidence interval [CI]: [−0.4% to 9.3%] and 3.0% 95% CI: [−2.7% to 8.6%], respectively.

**Conclusion:**

Progression of IF during the first year following kidney transplantation was not inferior among patients without CS compared with patients with CS.

## Graphical abstract

**Figure:**
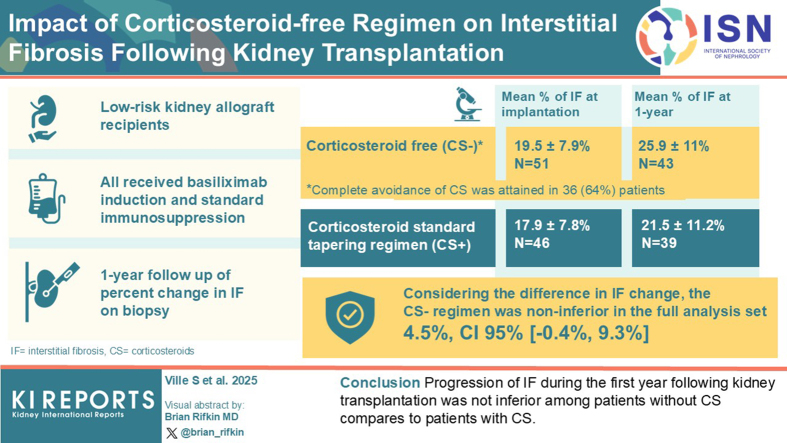

See Commentary on Page 2107

Kidney transplantation remains the standard treatment for patients suffering from kidney failure. In recent decades, the application of effective immunosuppressive regimens, combining calcineurin inhibitors, antimetabolite drugs, and CS as maintenance therapy associated with biological induction therapies, has resulted in a decreasing incidence of early T-cell–mediated rejection (TCMR). CS exposure, even at low dosage, can contribute to numerous side effects and adverse events, principally new-onset diabetes, dyslipidemia, hypertension, capillary fragility, cosmetic disorders, osteoporosis. We and others have demonstrated in randomized clinical trials (RCTs) that, in a low-immunological risk population, whatever the induction treatment, CS-free maintenance regimen (i.e., combining mycophenolate derivative and tacrolimus) did not increase the risk of clinical acute rejection,^1^, ^2^, ^3^, ^4^, ^5^, ^6^ while lowering the risk of CS-related adverse event.^6^, ^7^, ^8^, ^9^ Besides the risk of acute rejection, a crucial concern in CS-free regimens is the onset and progression of IF. This issue has been highlighted by studies addressing the natural history of the transplanted kidney through protocol biopsies that reported, even with living donors, that 60% of transplanted kidneys showed some degree of IF 1-year after transplantation. This IF was distributed between mild (representing between 10% and 25% of the cortex area) with a limited long-term consequence, or moderate (between 25% and 50%) which in contrast, negatively impacted graft survival. Part of this fibrosis is thought to be caused by ischemia-reperfusion occurring immediately after transplantation,^10^ and in the long run, to be driven by the calcineurin inhibitor toxicity. It has been assumed that both could be mitigated by CS,^2^^,^^11^, ^12^, ^13^ suggesting that a CS-free maintenance regimen will favor fibrosis progression during the first year following transplantation.

Although the Banff classification has allowed standardization of approaches to transplant histology, the semiquantitative variables have not been designed to monitor accurately process such as the fibrosis progression between 2 time points. For this purpose, validated tools assessing cortical fibrosis in a quantitative way (with continue variable) were developed.^14^ The major advantage of these methods is that it is automatized, therefore avoiding interobserved variations that have been reported with the Banff classification.^15^

The aim of the present RCT study was to address whether the absence of CS in kidney transplant recipients was associated with progression of the transplant fibrosis. IF was assessed at implantation, 3 months, and 1 year by an automated quantification method. In the light of recent advances that have pointed out the association between subclinical inflammation (including in the scarred area) and graft failure, we further investigated whether CS avoidance could favor such a process and potentially impact long-term survival.

## Methods

### Study Design

The study was an open, randomized, parallel-group, multicenter study with an active control and was conducted at 8 university hospital centers in France between April 2012 and April 2014 (NCT01541176). It was an institutional designed study (author, DC developed the original idea and is the principal investigator of the study; acronym name: ASTRONEF) and overall monitoring within centers was performed by the University Hospital of Nantes.

### Participants

Adult recipients (aged 18–70 years) of a kidney allograft from a non–human leukocyte antigen–identical living or deceased donor for whom a biopsy of the transplant kidney was performed at implantation, were eligible. Exclusion criteria were as follows: loss of previous kidney transplant because of rejection, positive T cell lymphocytotoxic crossmatch, history of a panel reactive antibody titer > 20%, positivity of donor-specific antibody assessed by Luminex, any combined transplantation, history of a previous solid organ transplantation, dual kidney transplantation, a donor whose heart was not beating at the time of organ harvest, donor receiving CS or other immunosuppressive drugs at the time of harvest, active hepatitis B or C, human immunodeficiency virus infection, and history of or evidence of cancer except keratinocyte cancer. All patients provided their written consent to participate in the study. The study was conducted in accordance with the Declaration of Helsinki, Declaration of Istanbul. Follow-up with of all patients was scheduled for 1 year.

### Intervention

Before the transplantation procedure, patients were randomly assigned in equal numbers to receive a CS-free regimen for primary maintenance immunosuppression (CS− group) or a standard CS tapering regimen (CS+ group) as follows: 20 mg of oral prednisone on day 2 through day 14, 15 mg on day 15 through day 30, 10 mg on day 31 through day 90, then from day 91, the dosage was maintained at 5 mg until postoperative day 365. All patients received induction therapy with 20 mg of basiliximab (Simulect, Novartis) on the day of surgery and day 4 and an i.v. bolus of 500 mg of methylprednisolone on the day of surgery. For both groups, maintenance immunosuppression was made up of 1 g of mycophenolate mofetil (CellCept, Roche) or mycophenolic acid 720 mg (Myfortic, Novartis) twice a day combined with tacrolimus as a once-daily extended-release formulation (Advagraf, Astellas), with a dosage chosen to achieve trough concentration ranges of serum levels of 8 to 12 ng/ml. Episodes of clinical rejection were histologically-proven and first-line treatment was with CS boluses.

### Renal Biopsy

Protocol biopsy was performed at baseline (implantation), 3 months, and 12 months following transplantation. Additional biopsy specimens were obtained in case of renal dysfunction. All biopsy specimens were examined locally (as well as centrally by authors KR and LHN) according to Banff 2013 criteria in a blinded fashion. Regarding the IF, results were expressed as percentage of IF and graded as follows: grade I: 10% to 25%, grade II: 25% to 50% and grade III > 50%. For protocol biopsy, IF was also assessed automatically as previously described.^14^ Briefly, the images were acquired using the 40× objective. Images of the entire cortex of the biopsy were obtained. Cortex was defined as the part inside the renal capsule and outside the medulla. The medulla was eliminated at the acquisition phase or during analysis by the observer. For each biopsy, the entire cortical region was analyzed in a stepwise fashion as a series of consecutive fields. Images were analyzed by a program of color segmentation image analysis. We used clustering techniques and particularly color image quantization. The program automatically extracts green color areas characteristic of IF. Renal capsule, tubular basement membranes, and sclerotic glomeruli were recognized and automatically excluded from analysis. Tubular membranes were removed from the green pixels according to their thickness. Capsule was recognized by its color and its location and sclerotic glomeruli were detected on their shape. The proportion of green to nongreen pixels in the image was calculated and used as an index of IF.

### Outcomes

The primary outcome was the difference in the percentage change of IF between the biopsy made at implantation and at 1-year, assessed by automatic quantification. The secondary outcomes were the change in IF between the biopsy made at implantation and at 1-year assessed by semiquantitative analysis (Banff classification), the incidence of delayed graft function, the incidence of acute rejection, the incidence of donor-specific antibody, the 1-year estimated renal function according to the Modification Diet in Renal Disease equation, and the incidence of graft failure. The 1-year posttransplantation incidence of adverse events was also evaluated, especially new-onset diabetes, dyslipidemia, hypertension, cytomegalovirus disease, and BK virus nephropathy.

### Statistical Analysis

The primary outcome measure was the between-group difference in fibrosis rate change in the group not receiving prolonged CS therapy and the group receiving systematic CS treatment at month 12, with a 95% CI in the PP and FAS populations. A noninferiority margin of 10% was selected so nonsuperiority will be demonstrated if the upper limit of the CI is no greater than 10%. The power calculation showed that a total of 186 patients would need to be included (i.e., 93 patients per group, assuming an SD of 20%, an initial risk of 2.5% [1-sided], 90% power, and a rate of patients not evaluable for the primary end point of 10% [loss mainly because of biopsy at 1 year and drop-outs]). Comparisons between categorical variables were made using the chi-square test, or Fisher Exact test, if necessary. For continuous variables, comparisons were performed using an unpaired 2-tailed *t* test. For survival and survival free from severe infection analysis, we achieved Kaplan–Meier curves and log-rank test using the R packages survival and survminer with R studio RStudio 2022.12 Elsbeth Geranium. *P*values < 0.05 were considered significant.

## Results

### Characteristics of the Patients

**Figure 1:**
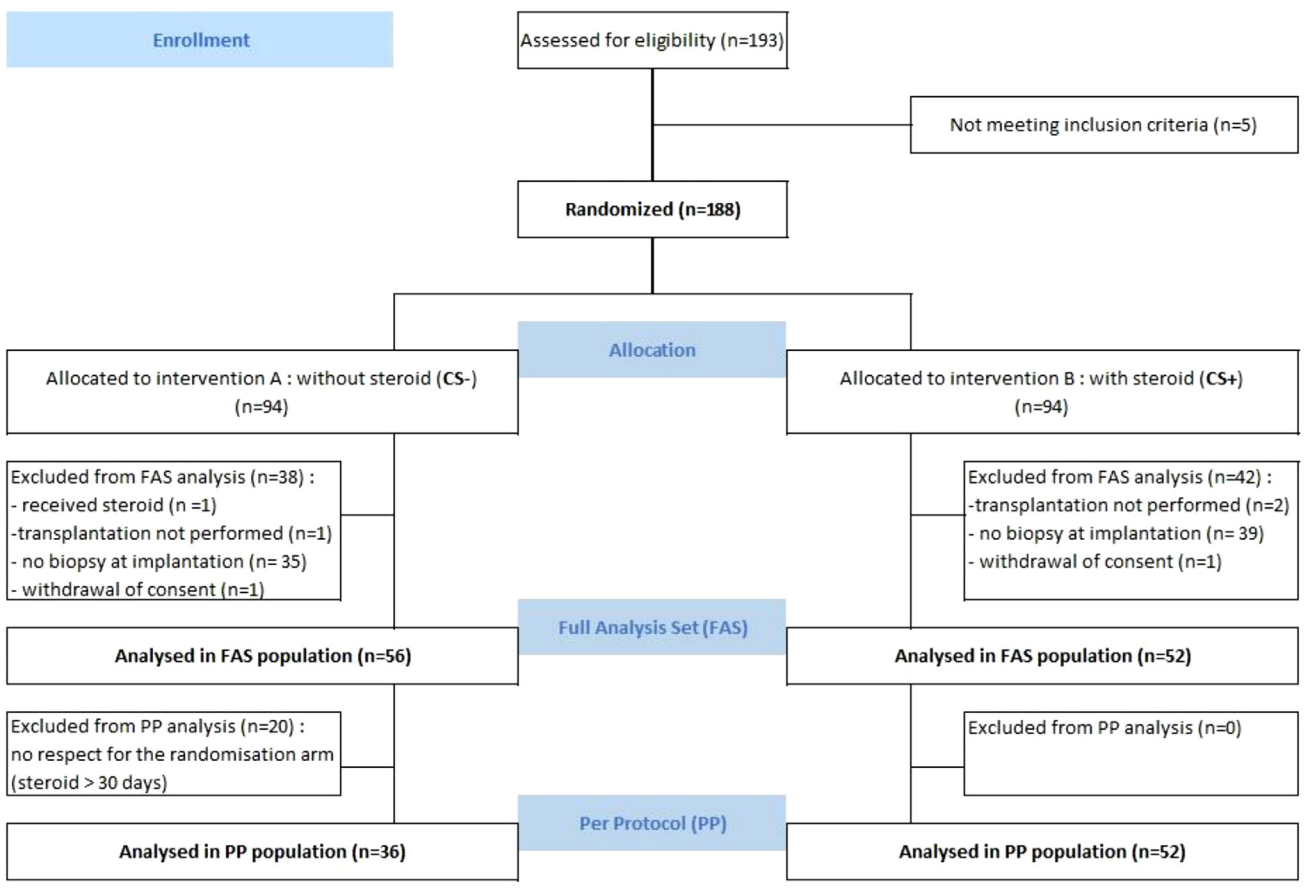
CONSORT flowchart of the study.

As illustrated in Figure 1, a total of 193 patients were enrolled and 188 randomized. Biopsy at implantation was not performed in 74 patients who were consequently not included in the analyses; 6 patients were excluded for other reasons. Eventually, 108 patients were analyzed in the FAS population: 52 patients in the CS+ group and 56 patients in the CS− group. The randomization procedure resulted in a well-balanced distribution of patients into 2 groups (Table 1 and Supplementary Table S1). The mean age of participants was 51 ± 1 years; 72% were male. CS were administered in CS− patients as part of first-line antirejection treatment in 8 patients and 12 others received transient CS treatment (> 30 d) for other reasons during the first year follow-up (to compensate the discontinuation of mycophenolate mofetil or mycophenolic acid [because of leucopenia, n = 5 or cytomegalovirus disease, *n* = 1]; adrenal insufficiency, n=1; and not specified, n=5). Overall, complete avoidance of CS was reached in 36CS− patients (64%), that make up, along with all 52 CS+ patients, the PP population. Tacrolimus trough levels were not different between the 2 groups (Supplementary Figure S1). With regard to mycophenolate mofetil or mycophenolic acid, patients in the CS− group received slightly higher doses between months 5 and 6 (1035 mg and 1020 mg vs. 840 and 830 mg mycophenolic acid dose equivalent, *P* < 0.05) (Supplementary Figure S2).

**Table 1:** Baseline demographic and clinical characteristics of the patients

Characteristics	CS+ (*n* = 52)	CS− (*n* = 56)	Total (*N* = 108)
Age (mean [SD]), yrs	50.3 (13.7)	52.1 (12.8)	51.3 (13.2)
Sex (%)			
Male	39 (75)	39 (70)	78 (72)
Female	13 (25)	17 (30)	30 (28)
Donor age (mean [SD])	50.0 (16.4)	54.2 (14.5)	52.1 (15.5)
Donor type (%)			
Living	3 ( 6)	5 ( 9)	8 ( 7)
Brain death	49 (94)	51 (91)	100 (93)
Death cause (donor) (%)			
Traumatic	9 (18)	6 (12)	15 (15)
Stroke	24 (49)	29 (57)	53 (53)
Other	16 (33)	16 (31)	32 (32)
Donor hypertension history (%)			
No	38 (73)	40 (71)	78 (72)
Yes	11 (21)	14 (25)	25 (23)
NA	3 (6)	2 (4)	5 (5)
Initial nephropathy (%)			
Other	24 (46)	26 (46)	50 (46)
Glomerulopathy	6 (11)	6 (11)	12 (11)
Tubulointerstitial or uropathy	3 (6)	3 (5)	6 (6)
Vascular	5 (10)	4 (7)	9 (8)
ADPKD	9 (17)	10 (18)	19 (18)
Diabetes	5 (10)	7 (12)	12 (11)
HLA mismatch (%)			
0	1 (2)	0 (0)	1 (1)
1–2	4 (9)	5 (12)	9 (10)
3–4	25 (57)	24 (55)	49 (56)
5–6	14 (32)	14 (33)	28 (32)
NA	8	13	21
Panel reactive antibody titer (%)			
0	37 (86)	39 (93)	76 (89)
1–15	6 (14)	3 (7)	9 (11)
NA	9	14	23
CMV status (%)			
D+/R+	16 (31)	15 (27)	31 (29)
D+/R-	11 (22)	16 (29.)	27 (25)
D−/R+	9 (18)	8 (14)	17 (16)
D−/R-	15 (29)	16 (29)	31 (29)
Cold ischemia time			
Min–Max	[0.8–23.5]	[0.4–23.6]	[0.4–23.6]
Mean (SD)	13.5 (5.0)	12.8 (6.2)	13.1 (5.7)

ADPKD, autosomal dominant polycystic kidney disease; CMV, cytomegalovirus; HLA, human leukocyte antigen.

### Change in IF Between Biopsy at Implantation and 1-Year Biopsy

**Figure 2:**
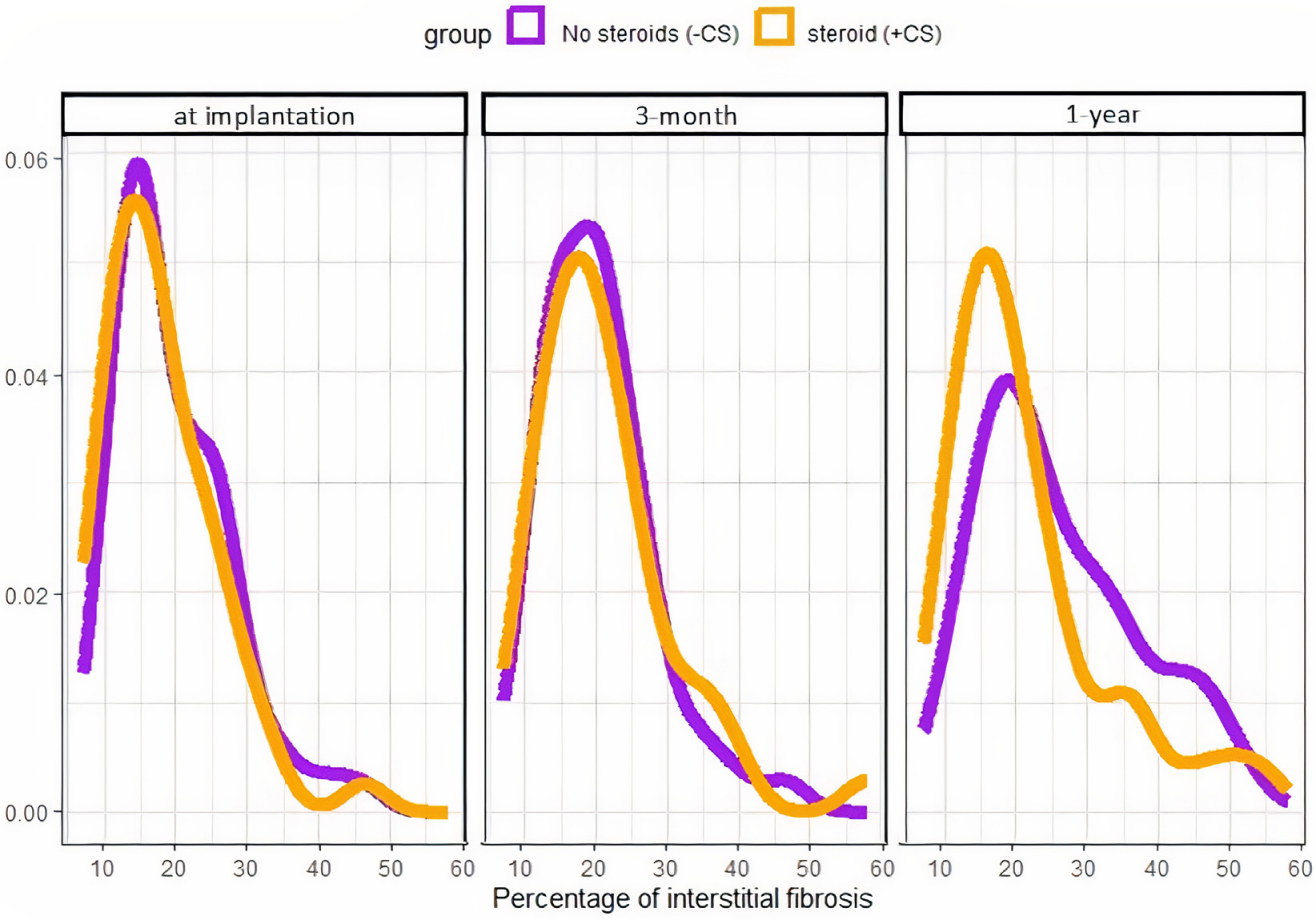
Percentage of cortical interstitial fibrosis assessed by automatic quantification at implantation and protocol biopsies, according to the treatment group (full analysis set population, *n* = 274 biopsies).

After the exclusion of inadequate biopsies, IF was assessed by using automatic quantification in 101 at implantation, 91 at 3 months and 82 at 1 year (Table 2). In Figure 2, we illustrate the distribution of the percentage of IF at each time point according to the treatment group. In the FAS population, mean percentage of IF at implantation was 19.5% ± 7.9% in the CS− group (*n* = 51) and 17.9% ± 7.8% in the CS+ group (*n* = 49; *P* = 0.3); at 3 months, it was 20.3% ± 7.8% (*n* = 49) in the CS− group and 21.1% ± 9.6% in the CS+ group (*n* = 42; *P* = 1); and at 1 year, it was 25.9% ± 11% (*n* = 43) in the CS− group and 21.5% ± 11.2% in the CS+ group (*n* = 39; *P* = 0.03). The progression of IF between the biopsy at implantation and at 1 year after transplantation was 7% ± 13.1% (*n* = 42) and 4.2% ± 11.5% (*n* = 37) in the CS− group and CS+ group, respectively (*P* = 0.3). When considering the difference, the CS-free regimen was noninferior 4.45%, 95% CI: [−0.4% to 9.3%]. In the PP population, the progression of IF between the biopsy at implantation and 1-year in the CS− group was 4.2% ± 12.8%, again noninferior 3.0%, 95% CI: [−2.7% to 8.6%].

**Table 2:** Interstitial fibrosis assessed by using quantitative automated quantification or semiquantitative quantification, at implantation and 3-months and 1-year protocol biopsies according to the treatment group, in the full analysis set and PP population

Characteristics	CS+ *n* = 52	FAS population		PP population	
		CS− *n* = 56	p-value	CS− *n* = 36	*P*-value
IF on biopsy at implantation (%)	17.9 (7.8)	19.5 (7.9)	0.3	20.8 (8.4)	0.078
NA	2	5		5	
IF on 3-month protocol biopsy (%)	21.1 (9.6)	20.3 (7.8)	>0.9	19.9 (7.2)	0.9
NA	10	7		6	
IF on 1-yr protocol biopsy (%)	21.5 (11.2)	25.9 (11.0)	0.031	24.4 (11.6)	0.2
NA	13	13		9	
Percentage change between implantation and 1-yr biopsies	4.2 (11.5)	7.0 (13.1)	0.3	4.2 (12.8)	>0.9
NA	15	14		10	
CI class on biopsy at implantation			>0.9		>0.9
0–1	45 (98%)	44 (98%)		26 (96%)	
2–3	1 (2.2%)	1 (2.2%)		1 (3.7%)	
NA	6	11		9	
CI class on 3-mos protocol biopsy			>0.9		0.7
0–1	38 (93%)	42 (91%)		25 (89%)	
2–3	3 (7.3%)	4 (8.7%)		3 (11%)	
NA	11	10		8	
CI class on 1-yr protocol biopsy			0.012		0.2
0-1	35 (88%)	26 (63%)		20 (74%)	
2-3	5 (12%)	15 (37%)		7 (26%)	
NA	12	15		9	

CI, confidence interval; CS, corticosteroids; FAS, full analysis set; IF, interstitial fibrosis; NA, not available; PP, per protocol.

**Figure 3:**
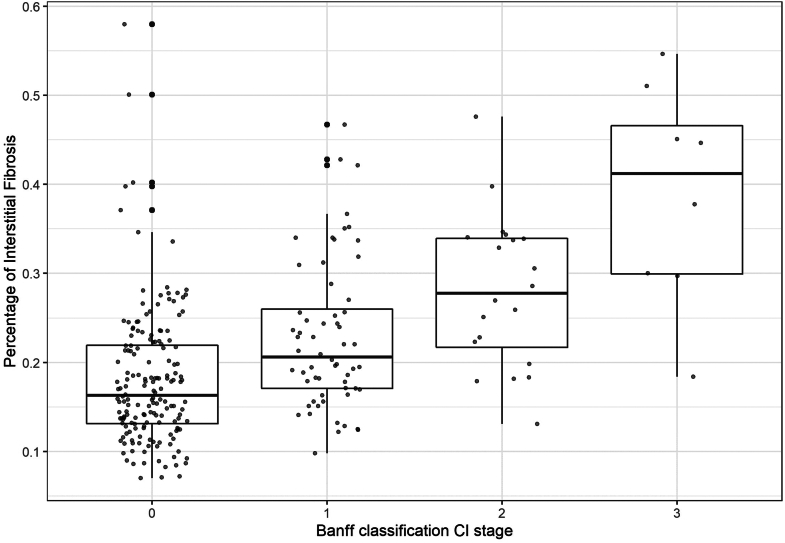
Correlation between semiquantitative assessment (Banff classification ci parameter) and automated quantitative quantification (Full Analysis Set population, *n* = 260 biopsies).

IF was also assessed in a semiquantitative way according to the scale defined by the Banff classification. In Figure 3, we illustrate the correspondence between the Banff scoring system and the automatic continuous quantification. In the FAS population, at implantation only 2.2% of biopsies showed a moderate to severe IF (ci 2 or 3) in both groups (CS−, *n* = 45; CS+, *n* = 46; *P* = 1), 7.3% and 8.7% at 3 months (CS−, *n* = 46, CS+, *n* = 41, *P* = 1) and finally, 37% and 12% at 1 year (CS−, *n* = 41; CS+, *n* = 40, *P* = 0.01). If we considered the CS− group in the PP population, at implantation, 3.7% of biopsies (*n* = 27, *P* = 1) showed a moderate to severe IF, 11% at 3 months (*n* = 28, *P* = 0.7) and 27% at 1 year (*n* = 27; *P* = 0.2).

### Secondary Outcomes

**Figure 4:**
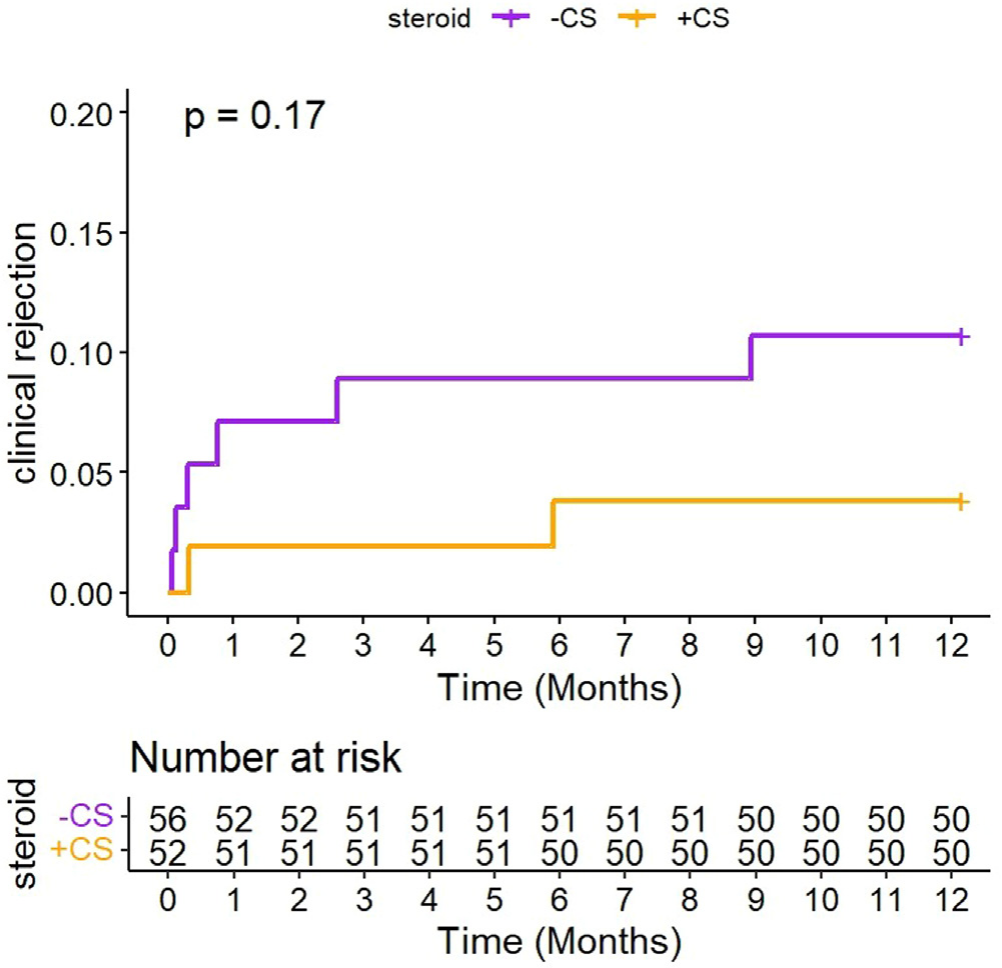
Log-rank curve representing the time until first clinical biopsy-proven acute rejection or borderline rejection (full analysis set).

As illustrated in Figure 4 and Supplementary Table S2, 1 year after transplantation, biopsy-proven acute rejection occurred in 4 patients (7.1%) in the CS− group and 2 (3.8%) in the CS+ group (*P* = 0.7). In all cases, it was a TCMR, 2 grade 1b and 2 grade 2a in the CS− group, and 2 grade 2a in the CS+ group. One patient in the CS+ experienced a recurrence. When borderline changes were included, the incidence of rejection was comparable between both groups (*n* = 6 [11%] in CS−; and *n* = 2 [3.8%] in CS+; *P* = 0.3). The rate of donor-specific antibody positivity by 1 year posttransplantation was nonsignificantly different (*n* = 2 [3.6%] in CS− and n=2 [3.6%] in CS+, *P* = 0.92) (Supplementary Table S3).

The need for dialysis (independently of the cause and number of sessions) during the posttransplant period was considered as delayed graft function. Delayed graft function occurred in 18 CS− patients (32%) and in 11 CS+ (22%) ones (*P* = 0.20).

The average glomerular filtration rate calculated by the Modification Diet in Renal Disease formula, the average of ratio of proteinuria-to-creatininuria, and the percentage of graft failure during the first year of transplantation did not differ significantly when considering the FAS population as well as the PP population (Supplementary Tables S4–S6).

In Supplementary Tables S7 to S10, we present the incidence of main adverse events according to CS exposure in the FAS. There was no significant difference regarding the safety of both regimens during the 12 months following transplantation regarding hypertension, new-onset diabetes after transplantation, dyslipidemia, cytomegalovirus, and BK positivity.

### Subclinical Inflammation in Protocol Biopsies

**Figure 5:**
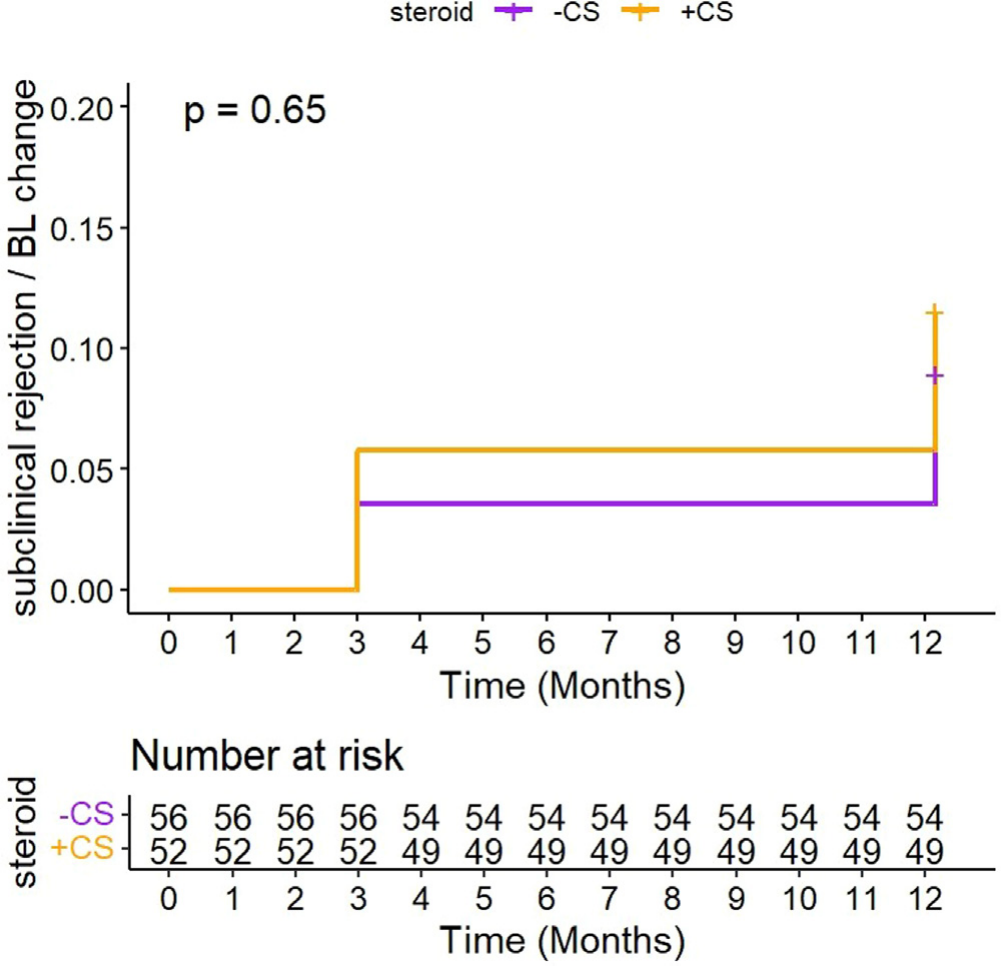
Log-rank curve representing the time until first subclinical biopsy-proven acute rejection or borderline rejection (full analysis set).

As illustrated in Figure 5, the incidence of the subclinical inflammation at 3-month and 12-month protocol biopsies was not different between the 2 groups. Subclinical TCMR occurred in 4 (patients 7.1%) in the CS− group and 3 (5.8%) in the CS+ group (*P* = 0.9). Subclinical borderline changes were observed in 1 patient (1.8%) in the CS− group and 3 (5.8%) in the CS+ group (*P* = 0.3). Of note the 2 biopsies that fulfilled the criteria for chronic active TCMR (t ≥ 2, total inflammation ≥ 2, i- interstitial fibrosis and tubular atrophy [IFTA] ≥ 2) also had acute TCMR. Examination of the extent of the cellular infiltration in the nonscarred area (Banff i), the scarred area (Banff i-IFTA) as well as the whole interstitial area (Banff ti), at 3-month and 12-month protocol biopsies did not shown significant difference between the 2 groups, considering either the FAS or the PP population for the CS− group (Table 3).

**Table 3:** Comparison of inflammation in 3-month and 1-year protocol biopsies according to the treatment group, in the full analysis set and PP population

Characteristics	3-mos protocolar biopsy					12-mos protocolar biopsy				
	CS+ *n* = 52	CS− FAS *n* = 56	*P*	CS+ PP *n* = 36	*P*	CS+ *n* = 52	CS− FAS *n* = 56	*P*	CS− PP *n* = 36	*p*
Banff i class (%)			0.7		0.3			>0.9		>0.9
0–1	39 (93)	44 (96)		28 (100)		39 (98)	39 (95)		26 (96)	
2–3	3 (7.1)	2 (4.3)		0 (0)		1 (2.5)	2 (4.9)		1 (3.7)	
NA	10	10		8		11	15		9	
Banff ti class (%)			0.6		0.5			>0.9		>0.9
0–1	39 (95)	45 (98)		28 (100)		38 (95)	38 (95)		25 (96)	
2–3	2 (4.9)	1 (2.2)		0 (0)		2 (5.0)	2 (5.0)		1 (3.8)	
NA	11	10		8		11	16		10	
Banff i-IFTA class (%)			0.5		0.4			0.3		0.13
0–1	38 (93)	40 (87)		24 (86)		33 (82)	37 (92)		26 (96)	
2–3	3 (7.3)	6 (13)		4 (14)		7 (18)	3 (7.5)		1 (3.7)	
NA	11	10		8		11	16		9	

CS, corticosteroids; FAS, full analysis set; IFTA, interstitial fibrosis and tubular atrophy; NA, not available; PP, per protocol.

### Long-Term Clinical Outcome

**Figure 6:**
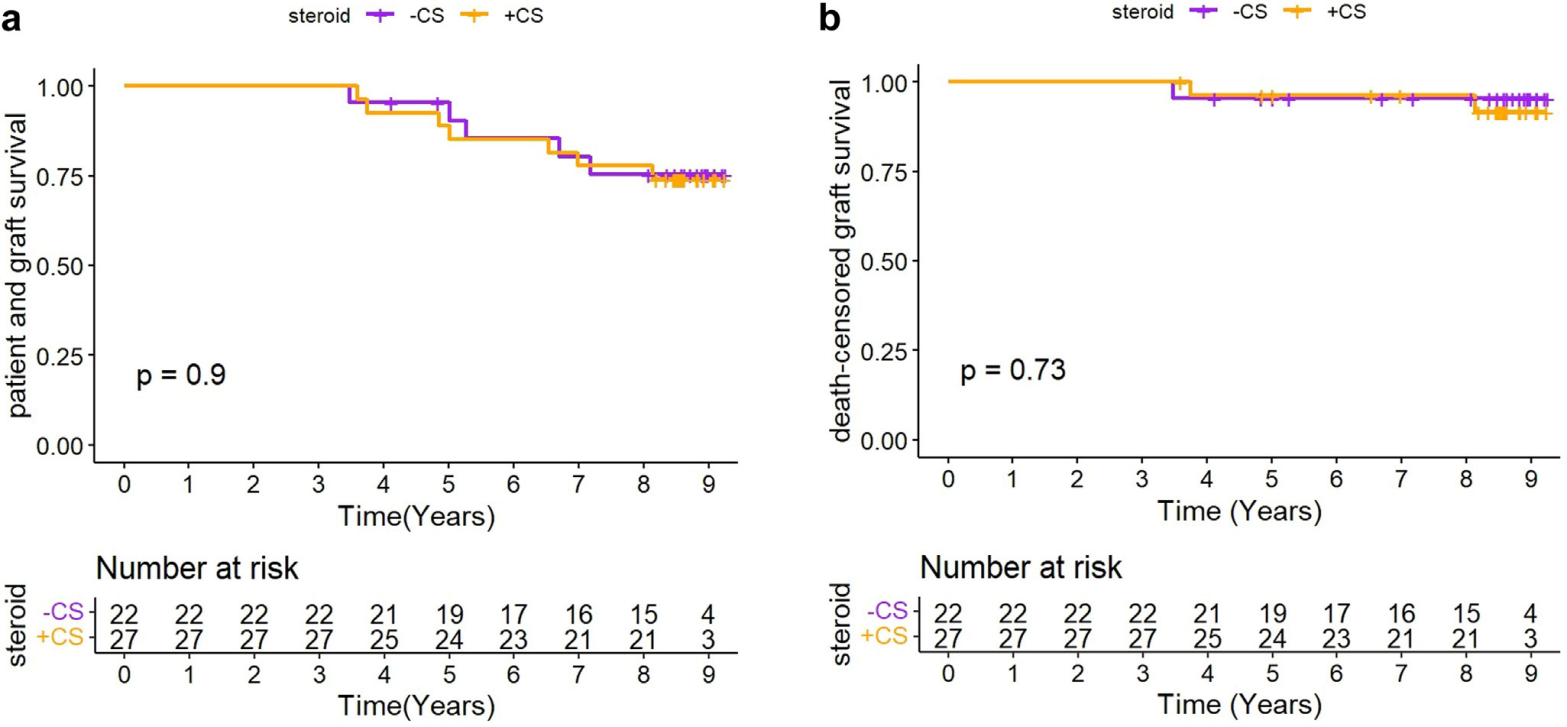
Crude survival curves estimated by the Kaplan–Meier estimator of population subgroup. (full analysis set). (a) Patient and graft survival. (b) Graft survival (death-censored).

In a subgroup of 46 patients (21 CS− and 25 CS+), corresponding to a particular center, we were able to evaluate the long-term outcome (mean follow-up 3036 days, range: 1270–3377). No difference between the 2 groups was observed, with 5-year death-censured graft survival of 95% and 96%, and 5-year patient survival of 90% and 85%, in the CS− and CS+ groups, respectively (Figure 6a and b).

## Discussion

In this randomized multicenter clinical trial, we found that the progression of fibrosis, assessed by a quantitative automated method, was not inferior (considering a 10% noninferiority margin) in recipients receiving or not receiving CS throughout the first postoperative year after kidney transplantation.

Numerous RCTs of CS avoidance or rapid CS withdrawal had until now evaluated the safety regarding the risk of clinical acute rejection as well as the potential to reduce adverse events.^16^ Our study is the first to have addressed specifically the question of whether CS avoidance could impact the histopathological progression of IF measured by a quantitative automated method during the first year after surgery. To date, 2 RCTs investigated this issue using conventional semiqualitative assessment methods. In both studies (and as in our study), patients received induction treatment and tacrolimus. In the first one, conducted in 60 kidney transplant recipients with serial protocol biopsies at 1, 6, and 12 months, a trend toward more fibrosis at 1 year in the group without CS was noted, despite no difference in terms of rejection rate. It was concluded that CS might prevent calcineurin inhibitor–induced fibrosis.^2^ In the second study, carried out in a pediatric patient population of kidney transplant recipients,^17^ no difference regarding the children’s growth and the incidence of acute rejection was noted. The authors also scrutinized the chronic allograft damage index in serial protocol biopsies performed at 6, 12, and 24 months after transplantation and did not find any difference between treatment groups.^18^ In our study, though the noninferiority hypothesis was confirmed, deeper analyses were made possible because of the accuracy of the fibrosis quantitative assessment. When we focused on the early period following transplantation, we found that although not significantly different, the progression of IF from the time of implantation to 3 months was higher in the CS+ group than in the CS− group (3.8% ± 8.6% vs. 1.5% ± 9.5%). These results are in line with recent meaningful data having contradicted the idea that argued for a beneficial effect of CS in the early stage of kidney transplantation. Indeed, though ischemia-reperfusion injury following kidney transplantation was thought to induce inflammation and subsequent fibrosis,^11^ a retrospective study that analyzed at set of biopsies within the first 2 weeks after transplantation, did not find any association between cold ischemia time, donor age, donor type and inflammatory changes.^19^ Similarly, delayed graft function, a recognized risk-factor for ischemia-reperfusion injury, was neither associated with the progression of fibrosis during the first year after transplantation.^19^

When we closely scrutinized the period 3 months to 12-month, the percentage of fibrosis did not change in the FAS and PP CS+ group (from 21.1 ± 9.6 to 21.5 ± 11.2, *P* = 0.8) whereas a significant increase was observed in the FAS CS− group (20.3 ± 7.8 to 25.9 ± 11, *P* = 0.02) but not in the PP one (19.9 ± 7.2 to 24.4 ± 11.6, *P* = 0.3). In Figure 2, we highlight this discreet shift. Whether CS could mitigate calcineurin inhibitor–induced fibrosis is a matter of controversies.^13^ As often after kidney transplantation, the allo-immune response should always be suspected in the pathogenesis of histological lesions. We observed a very low rate of clinical acute rejection or borderline lesions. This observation is concordant with that of the HARMONY trial.^6^ In this RCT study focused on a rapid CS withdrawal regimen, a low rate of biopsy-proven acute rejection (∼10%) was observed independent of the CS regimen. The association of systematic induction and tacrolimus maintenance immunosuppression (same as our study) was considered to be the main explanation. This immunosuppressive regimen is now considered by many as gold standard reference. This regimen could have changed the clinical and pathological picture of the rejection, evolving from a full-scale to a more partial and latent presentation. Beyond the now well-known involvement of the humoral response in late graft failure, serial protocol biopsies studies have also demonstrated that IFTA progression, ending up in graft failure, was also associated with early subclinical IF.^20^, ^21^, ^22^, ^23^, ^24^ These studies further highlighted the importance of taking in account the infiltration located in the scarred area,^25^^,^^26^ that have been recognized in the last Banff meeting report, because i-IFTA and total inflammation and are now part of the chronic active TCMR diagnosis.^27^

To investigate whether CS avoidance could favor such subclinical inflammation, we further evaluate subclinical TCMR and borderline rejection (Figure 5) as well as Banff lesions associated with interstitial inflammation (i, i-IFTA, and total inflammation) in 3-month and 1-year protocol biopsies (Table 3). We did not observe any difference between the 2 groups, whichever population was considered (whether FAS or PP), suggesting that the slight increase in fibrosis observed in the CS− group was not driven by an ongoing latent T-cell response. To determine whether this difference negatively impacted long-term outcome, death-censored graft survival was evaluated in a subset of 46 patients in our center that were followed-up with prospectively. There was no difference in graft survival between CS− and CS+ recipients at 5 years and even further. Thus, the small shift in IF that we observed in CS patients does not appear to be clinically significant, although our data must be interpreted with caution because it represents a *post hoc* analysis in a fraction of patients. Recent studies in fact demonstrate that mild fibrosis in 1-year protocol biopsy (< 25%; i.e., the mean percentage assessed in our CS− group) did not impact graft survival when isolated; meaning, without significant inflammation in unscarred or scarred area.^22^^,^^25^^,^^26^^,^^28^

Our study has some evident limitations. Mostly, a large proportion of patients initially randomized had to be excluded from the analysis because of the absence (or bad quality of the specimen) of biopsy at implantation. As a result, we assessed 108 patients, of whom 79 had reliable data to analyze the primary outcome, though we had planned to enroll 186 patients to analyze 170. Although we found that the 4.45% difference by steroid exposure for fibrosis change was noninferior based on the prespecified noninferiority margin of 10%, the upper limit of the CI was close to 10% (9.3%). Therefore, our results should be interpreted with caution and should not be considered as proof of equivalence between the 2 regimens. A larger study would have provided greater statistical power and more precise estimates of treatment effects. Perhaps because of the unblinded design, the dose of antiproliferative drugs was higher in the group without steroids; this difference, however small, may have affected the results. Besides the innovative monitoring of fibrosis used in our study, other approaches such as the molecular microscope diagnostic system,^29^ would have been valuable to deeper evaluate the potential detrimental immunological impact of CS avoidance. Unfortunately, our study was not designed to test this methodology. No specific scale was used besides classical parameters, such as infection and diabetes, to capture CS toxicity; such a tool may have made it possible to further appreciate the benefits of CS avoidance. Because not initially intended, long-term follow-up, beyond the first year was partial and should be regarded with caution. Only patients considered at low-immunological risk were included and other studies are due to extend our rather reassuring results to patients at high-risk that made up a large fraction of kidney transplant recipients.

In conclusion, the difference in fibrosis change during the first transplant year assessed by automated continuous quantification was noninferior considering a noninferiority margin of 10%, whether steroids were used or not. In addition, neither clinical (rejection) nor subclinical (inflammation of untouched or scarred areas) allo-immune responses, nor long-term graft survival differed with CS use.

## Disclosure

LR received payment for presentations from Astellas, BMS, and Sanofi. NK received funding from Astellas; consulting fees from Biotest, BMS, Chiesi, ExeViR, Grifols, Hansa, MSD, Synklino, and Takeda; payment for presentations from Astellas, Biotest, BMS, CSL Behring, Chiesi, Gilead, Grifols, Hansa, MSD, Glasgow Smith Kline, Pierre Fabre, Medison, Neovii, Roche, Sanofi, Sandoz, and Takeda; support for attending meetings and/or travel from Astellas, Biotest, BMS, CSL Behring, Chiesi, Gilead, Grifols, Hansa, MSD, Glasgow Smith Kline, Pierre Fabre, Medison, Neovii, Roche, Sanofi, Sandoz, and Takeda; and participation on a Data Safety Monitoring Board or Advisory Board from Alexion, Astellas, AstraZeneca, Biotest, BMS, Chiesi, ExeViR, Grifols, Hansa, MSD, Synklino, and Takeda. SV received payment for presentations from Novartis, CSL Vifor, Alexion, and Sobi; and support for attending meetings and/or travel from Astellas and Chiesi. All the other authors declared no competing interests.

## Supplementary Material

Supplementary File (PDF) **Figure S1.** Mean tacrolimus trough level according to the treatment group. **Figure S2.** Mean antiproliferative drugs doses (mg eq. mycophenolic acid). **Table S1.** Comparison between the patients of the FAS population (*n* = 108) and the n = 80 patients excluded from the FAS analysis after the randomization mostly because no biopsy at implantation was made. **Table S2.** Incidence of acute rejection and borderline change on for-cause and protocolar biopsy. **Table S3.** Incidence of donor-specific antibody. **Table S4.** Glomerular filtration rate (according to the Modification Diet in Renal Disease formula). **Table S5.** Urine protein-to-creatinine ratio (mmol/mg). Table S6. Graft failure. **Table S7.** Adverse event, hypertension. **Table S8.** Adverse event, dyslipidemia. **Table S9.** Adverse event, new-onset diabetes after transplantation. **Table S10.** Viral reactivation.

## Footnotes

Supplementary File (PDF)
**Figure S1.** Mean tacrolimus trough level according to the treatment group. **Figure S2.** Mean antiproliferative drugs doses (mg eq. mycophenolic acid). **Table S1.** Comparison between the patients of the FAS population (*n* = 108) and the n = 80 patients excluded from the FAS analysis after the randomization mostly because no biopsy at implantation was made. **Table S2.** Incidence of acute rejection and borderline change on for-cause and protocolar biopsy. **Table S3.** Incidence of donor-specific antibody. **Table S4.** Glomerular filtration rate (according to the Modification Diet in Renal Disease formula). **Table S5.** Urine protein-to-creatinine ratio (mmol/mg). **Table S6.** Graft failure. **Table S7.** Adverse event, hypertension. **Table S8.** Adverse event, dyslipidemia. **Table S9.** Adverse event, new-onset diabetes after transplantation. **Table S10.** Viral reactivation.
