# Supplementary material for: Impact of Corticosteroid-Free Regimen on Interstitial Fibrosis Following Kidney Transplantation
Source: Kidney Int Rep. 2025 Apr 6;10(7):2222–32. doi: 10.1016/j.ekir.2025.04.004 (PMC12266256; doi:10.1016/j.ekir.2025.04.004)
Supplement: Supplementary File (PDF) — Figure S1. Mean tacrolimus trough level according to the treatment group. Figure S2. Mean antiproliferative drugs doses (mg eq. mycophenolic acid). Table S1. Comparison between the patients of the FAS population (n = 108) and the n = 80 patients excluded from the FAS analysis after the randomization mostly because no biopsy at implantation was made. Table S2. Incidence of acute rejection and borderline change on for-cause and protocolar biopsy. Table S3. Incidence of donor-specific antibody. Table S4. Glomerular filtration rate (according to the Modification Diet in Renal Disease formula). Table S5. Urine protein-to-creatinine ratio (mmol/mg). Table S6. Graft failure. Table S7. Adverse event, hypertension. Table S8. Adverse event, dyslipidemia. Table S9. Adverse event, new-onset diabetes after transplantation. Table S10. Viral reactivation. [file mmc1.pdf]

Supplemental Tables

Supplemental Table 1, comparison between the patients of the FAS population (n=108) and the n=80 patients excluded from the FAS analysis after the randomisation mostly because no biopsy at implantation was made.

		FAS population (n = 108)	No biopsy at implantation (n=80)	FAS population with M12 biopsy	Total N=188
Age (mean (SD))		51.2(13,2)	52.6(13.9)	50.1(12.0)	51.8(13.5)
Sex (%)	Male	78 (72)	57 (71)	59(69)	135 (72)
	Female	30 (28)	23 (29)	21(31)	53 (28)
Donor Type (%)	Living	8 (7)	13 (17)	7(8)	21 (11)
	Brain death	100 (93)	65 (83)	79(92)	165 (89)
Donor age (mean (SD))		52.1(15.5)	51.8(15.2)	54.6(15.9)	53.2(15.7)
Death cause (donor) (%)	Traumatic	15 (15)	13 (20)	11(13)	28 (17)
	Stroke	55 (55)	33 (51)	46(53)	88 (53)
	Other	30 (30)	19 (29)	29(34)	49 (30)
Donor hypertension history (%)	No	78 (72)	37 (47)	65(76)	115 (62)
	Yes	25 (23)	24 (31)	17(20)	49 (26)
	NA	5(4)	17(22)	4(4)	22(12)
HLA mismatch (%)	0	1 (1)	2 (3)	1(2)	3 (2)
	1-2	9 (10)	5 (7)	9(14)	14 (9)
	3-4	49 (56)	39 (55)	33(51)	88 (56)
	5-6	28 (32)	25 (35)	22(34)	53 (33)
	NA	21	9	21	30
Panel Reactive Antibody titer (%)	0	76 (89)	45 (82)	64(88)	121 (86)
	1-15	9 (11)	10 (18)	9(12)	19 (14)
	NA	23	25	13	48
CMV status (%)	D+/R+	31 (29)	26 (34)	28(33)	57 (31)
	D+/R-	27 (25)	20 (26)	21(25)	47 (26)
	D-/R+	17 (16)	11 (14)	13(15)	28 (15)
	D-/R-	31 (29)	20 (26)	23(27)	51 (28)
Cold Ischiemia Time	Min-Max	[0.4;23.6]	[0.7;23.3]	[0.40;23.58]	[0.4;23.6]
	Mean (SD)	13.1(5.7)	12.5(6.4)	13.0(5.7)	12.9(6.0)

Supplemental Table 2, Incidence of acute rejection and borderline change on for cause and protocolar biopsy.

		CS-<br>N=56	CS+<br>N=52	Total<br>N=108
clinical rejection/borderline (for cause biopsy)		56	52	108
	TCMR	4 (7.1%)	2 (3.8%)	6 (5.5%)
	Borderline change	2(3.5%)	0	2(1.8%)
	Treatment (steroids alone/anti thymocyte globulin)	5/1	1/1	6/2
Subclinical (protocolar biopsy)				
	TCMR	4 (7.1%)	3 (5.8%)	7(6.4%)
	Borderline change	1 (1.8%)	3 (5.8%)	4(3.7%)

Supplemental Table 3, Incidence of DSA

		CS-<br>N=56	CS+<br>N=52	Total<br>N=108
DSA - M03	N	56	52	108
	Negative	55 (98.21%)	51 (98.08%)	106 (98.15%)
	Positive	1 (1.79%)	1 (1.92%)	2 (1.85%)
DSA - M03				
DSA - M12	N	56	52	108
	Negative	54 (96.43%)	50 (96.15%)	104 (96.30%)
	Positive	2 (3.57%)	2 (3.85%)	4 (3.70%)

Supplemental Table 4, Glomerular Filtration rate (according to the MDRD formula)

		CS-<br>N=56	CS+<br>N=52	Total<br>N=108
GFR - M3	Missing data	1	3	4
	N	55	49	104
	Min-Max	[16.60;106.00]	[16.78;99.80]	[16.60;106.00]
	Mean+/- SD	50.50+/-18.84	55.29+/-19.50	52.76+/-19.21
	Median[Q1;Q3]	50.99[36.60;62.71]	55.00[41.00;66.91]	52.12[39.00;64.35]
DFG - M03				
GFR - M6	Missing data	1	2	3
	N	55	50	105
	Min-Max	[18.10;115.00]	[18.70;109.14]	[18.10;115.00]
	Mean+/- SD	51.15+/-20.40	53.80+/-22.57	52.41+/-21.40

		CS-<br>N=56	CS+<br>N=52	Total<br>N=108
	Median[Q1;Q3]	49.00[35.26;65.80]	52.31[34.44;71.71]	50.00[34.77;66.19]
DFG - M06				
GFR - M12	Missing data	3	3	6
	N	53	49	102
	Min-Max	[5.80;95.88]	[19.10;114.60]	[5.80;114.60]
	Mean+/- SD	51.86+/-19.07	57.65+/-21.55	54.64+/-20.41
	Median[Q1;Q3]	50.00[42.00;64.60]	54.00[41.22;71.05]	52.28[42.00;66.45]

Supplemental Table 5, urine Protein Creatinine Ratio (mmol/mg)

		CS-<br>N=56	CS+<br>N=52	Total<br>N=108
uPCR (mg/mmol) - M03	Missing data	5	6	11
	N	51	46	97
	Min-Max	[0.00;156.00]	[0.00;808.00]	[0.00;808.00]
	Mean+/- SD	16.12+/-24.93	36.09+/-123.38	25.59+/-86.94
	Median[Q1;Q3]	10.00[0.01;22.90]	3.05[0.01;17.65]	8.10[0.01;21.28]
Rapport				
uPCR (mg/mmol) - M06	Missing data	7	4	11
	N	49	48	97
	Min-Max	[0.01;338.46]	[0.00;326.92]	[0.00;338.46]
	Mean+/- SD	23.72+/-53.52	39.88+/-77.12	31.72+/-66.41
	Median[Q1;Q3]	9.65[0.03;18.69]	8.67[0.01;33.85]	9.44[0.02;25.64]
Rapport				
uPCR (mg/mmol) - M12	Missing data	4	7	11
	N	52	45	97
	Min-Max	[0.00;214.55]	[0.00;360.38]	[0.00;360.38]
	Mean+/- SD	20.52+/-35.30	32.02+/-60.62	25.86+/-48.78
	Median[Q1;Q3]	12.48[0.02;22.50]	8.49[0.01;47.27]	10.00[0.01;24.79]

Supplemental Table 6, Graft failure

		CS-<br>N=56	CS+<br>N=52	Total<br>N=108
Graft failure	N	56	52	108
	No	55 (98.21%)	51 (98.08%)	106 (98.15%)
	Yes	1 (1.79%)	1 (1.92%)	2 (1.85%)

Supplemental Table 7, adverse event, hypertension

		**CS-<br>N=56**	**CS+<br>N=52**	**Total<br>N=108**
hypertension	N	56	52	108
	No	31 (55.36%)	29 (55.77%)	60 (55.56%)
	Yes	25 (44.64%)	23 (44.23%)	48 (44.44%)

Supplemental Table 8, adverse event, dyslipidemia

		**CS-<br>N=56**	**CS+<br>N=52**	**Total<br>N=108**
Dyslipidémie	N	56	52	108
	No	55 (98.21%)	47 (90.38%)	102 (94.44%)
	Yes	1 (1.79%)	5 (9.62%)	6 (5.56%)

Supplemental Table 9, adverse event, NODAT

		**CS-<br>N=56**	**CS+<br>N=52**	**Total<br>N=108**
NODAT	N	56	52	108
	No	47 (83.93%)	38 (73.08%)	85 (78.70%)
	Yes	9 (16.07%)	14 (26.92%)	23 (21.30%)

Supplemental Table 10, viral reactivation

		**CS-<br>N=56**	**CS+<br>N=52**	**Total<br>N=108**
CMV	N	56	52	108
	Negative	42 (75.00%)	41 (78.85%)	83 (76.85%)
	Positive	14 (25.00%)	11 (21.15%)	25 (23.15%)
CMV				
CMV with treatment	N	56	52	108
	No	48 (85.71%)	48 (92.31%)	96 (88.89%)
	Yes	8 (14.29%)	4 (7.69%)	12 (11.11%)
CMV traité				
BKV	N	56	52	108
	No	49 (87.50%)	47 (90.38%)	96 (88.89%)
	Yes	7 (12.50%)	5 (9.62%)	12 (11.11%)

Supplemental Figure

Supplemental Figure 1, mean tacrolimus trough level according to the treatment group.

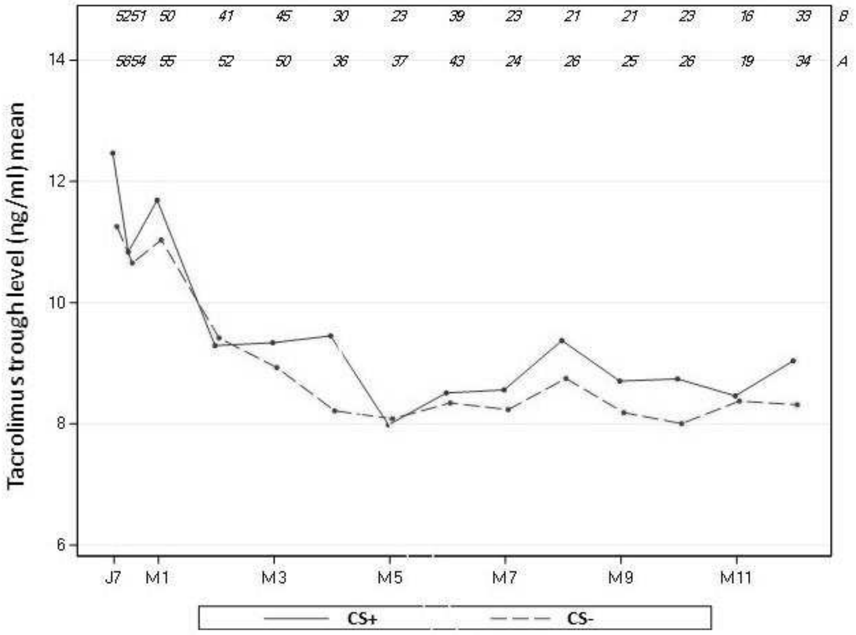

Supplemental Figure 2, mean antiproliferative drugs doses (mg eq. mycophenolic acid)

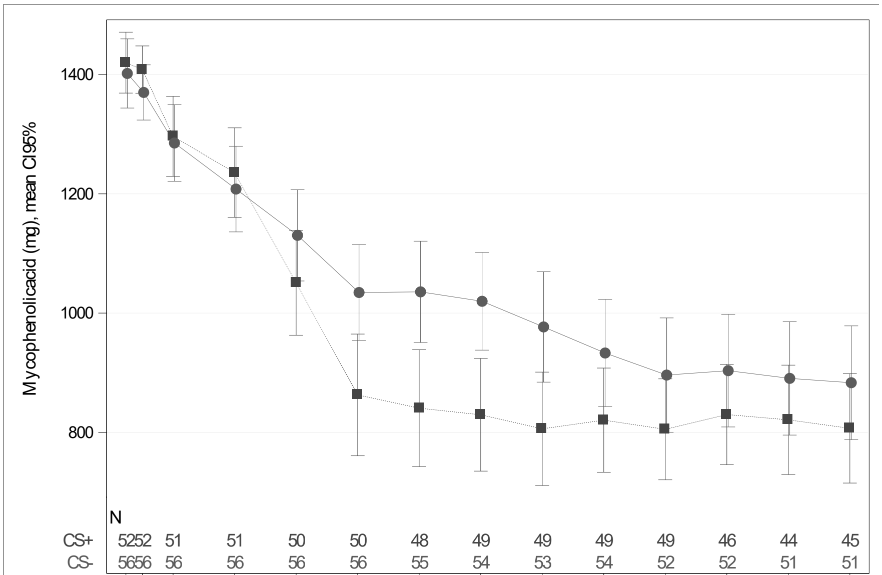

CONSORT 2010 checklist of information to include when reporting a randomised trial*

Section/Topic	Item No	Checklist item	Reported on page No
**Title and abstract**			
	1a	Identification as a randomised trial in the title	1
	1b	Structured summary of trial design, methods, results, and conclusions (for specific guidance see CONSORT for abstracts)	2
**Introduction**			
Background and objectives	2a	Scientific background and explanation of rationale	3
	2b	Specific objectives or hypotheses	3
**Methods**			
Trial design	3a	Description of trial design (such as parallel, factorial) including allocation ratio	4
	3b	Important changes to methods after trial commencement (such as eligibility criteria), with reasons	NA
Participants	4a	Eligibility criteria for participants	4
	4b	Settings and locations where the data were collected	4
Interventions	5	The interventions for each group with sufficient details to allow replication, including how and when they were actually administered	4
Outcomes	6a	Completely defined pre-specified primary and secondary outcome measures, including how and when they were assessed	5
	6b	Any changes to trial outcomes after the trial commenced, with reasons	NA
Sample size	7a	How sample size was determined	5
	7b	When applicable, explanation of any interim analyses and stopping guidelines	NA
Randomisation:			
Sequence generation	8a	Method used to generate the random allocation sequence	4
	8b	Type of randomisation; details of any restriction (such as blocking and block size)	4
Allocation concealment mechanism	9	Mechanism used to implement the random allocation sequence (such as sequentially numbered containers), describing any steps taken to conceal the sequence until interventions were assigned	4
Implementation	10	Who generated the random allocation sequence, who enrolled participants, and who assigned participants to interventions	4
Blinding	11a	If done, who was blinded after assignment to interventions (for example, participants, care providers, those	NA

		assessing outcomes) and how	
	11b	If relevant, description of the similarity of interventions	NA
Statistical methods	12a	Statistical methods used to compare groups for primary and secondary outcomes	5
	12b	Methods for additional analyses, such as subgroup analyses and adjusted analyses	5
**Results**			
Participant flow (a diagram is strongly recommended)	13a	For each group, the numbers of participants who were randomly assigned, received intended treatment, and were analysed for the primary outcome	Figure 1
	13b	For each group, losses and exclusions after randomisation, together with reasons	Figure 1
Recruitment	14a	Dates defining the periods of recruitment and follow-up	4
	14b	Why the trial ended or was stopped	NA
Baseline data	15	A table showing baseline demographic and clinical characteristics for each group	Table 1
Numbers analysed	16	For each group, number of participants (denominator) included in each analysis and whether the analysis was by original assigned groups	Figure 1
Outcomes and estimation	17a	For each primary and secondary outcome, results for each group, and the estimated effect size and its precision (such as 95% confidence interval)	6
	17b	For binary outcomes, presentation of both absolute and relative effect sizes is recommended	6
Ancillary analyses	18	Results of any other analyses performed, including subgroup analyses and adjusted analyses, distinguishing pre-specified from exploratory	7
Harms	19	All important harms or unintended effects in each group (for specific guidance see CONSORT for harms)	9
**Discussion**			
Limitations	20	Trial limitations, addressing sources of potential bias, imprecision, and, if relevant, multiplicity of analyses	9
Generalisability	21	Generalisability (external validity, applicability) of the trial findings	8-10
Interpretation	22	Interpretation consistent with results, balancing benefits and harms, and considering other relevant evidence	8-10
**Other information**			
Registration	23	Registration number and name of trial registry	4
Protocol	24	Where the full trial protocol can be accessed, if available	NA
Funding	25	Sources of funding and other support (such as supply of drugs), role of funders	11

Citation: Schulz KF, Altman DG, Moher D, for the CONSORT Group. CONSORT 2010 Statement: updated guidelines for reporting parallel group randomised trials. BMC Medicine. 2010;8:18.
© 2010 Schulz et al. This is an Open Access article distributed under the terms of the Creative Commons Attribution License (http://creativecommons.org/licenses/by/2.0), which permits unrestricted use, distribution, and reproduction in any medium, provided the original work is properly cited.

*We strongly recommend reading this statement in conjunction with the CONSORT 2010 Explanation and Elaboration for important clarifications on all the items. If relevant, we also recommend reading CONSORT extensions for cluster randomised trials, non-inferiority and equivalence trials, non-pharmacological treatments, herbal interventions, and pragmatic trials. Additional extensions are forthcoming: for those and for up-to-date references relevant to this checklist, see www.consort-statement.org.
